# Differential Detection of Encapsidated versus Unencapsidated Enterovirus RNA in Samples Containing Pancreatic Enzymes—Relevance for Diabetes Studies

**DOI:** 10.3390/v12070747

**Published:** 2020-07-11

**Authors:** Maarit Oikarinen, Lori Bertolet, Antonio Toniolo, Sami Oikarinen, Jutta E. Laiho, Alberto Pugliese, Richard E. Lloyd, Heikki Hyöty, the nPOD-V Study Group

**Affiliations:** 1 Faculty of Medicine and Health Technology, Tampere University, 33520 Tampere, Finland; sami.oikarinen@tuni.fi (S.O.); jutta.laiho@tuni.fi (J.E.L.); heikki.hyoty@tuni.fi (H.H.); 2 Department of Molecular Virology and Microbiology, Baylor College of Medicine, Houston, TX 77030, USA; llbertolet@mdanderson.org (L.B.); rlloyd@bcm.edu (R.E.L.); 3 Global Virus Network, University of Insubria, 21100 Varese, Italy; antonio.toniolo@gmail.com; 4 Diabetes Research Institute, Miller School of Medicine, University of Miami, Miami, FL 33136, USA; apuglies@med.miami.edu; 5 Fimlab Laboratories, Pirkanmaa Hospital District, 33520 Tampere, Finland

**Keywords:** enterovirus, virus detection, RT-PCR, pancreas, type 1 diabetes

## Abstract

Using immunohistochemistry, enterovirus capsid proteins were demonstrated in pancreatic islets of patients with type 1 diabetes. Virus proteins are mainly located in beta cells, supporting the hypothesis that enterovirus infections may contribute to the pathogenesis of type 1 diabetes. In samples of pancreatic tissue, enterovirus RNA was also detected, but in extremely small quantities and in a smaller proportion of cases compared to the enteroviral protein. Difficulties in detecting viral RNA could be due to the very small number of infected cells, the possible activity of PCR inhibitors, and the presence—during persistent infection—of the viral genome in unencapsidated forms. The aim of this study was twofold: (a) to examine if enzymes or other compounds in pancreatic tissue could affect the molecular detection of encapsidated vs. unencapsidated enterovirus forms, and (b) to compare the sensitivity of RT-PCR methods used in different laboratories. Dilutions of encapsidated and unencapsidated virus were spiked into human pancreas homogenate and analyzed by RT-PCR. Incubation of pancreatic homogenate on wet ice for 20 h did not influence the detection of encapsidated virus. In contrast, a 15-min incubation on wet ice dramatically reduced detection of unencapsidated forms of virus. PCR inhibitors could not be found in pancreatic extract. The results show that components in the pancreas homogenate may selectively affect the detection of unencapsidated forms of enterovirus. This may lead to difficulties in diagnosing persisting enterovirus infection in the pancreas of patients with type 1 diabetes.

## 1. Introduction

Enteroviruses (EVs) are linked to the pathogenesis of type 1 diabetes (T1D). Epidemiological studies showed a risk association between EV infections and the development of islet autoimmunity and T1D, and recent reports indicated that a low-level EV infection may occur in the pancreata of patients with recent onset T1D [1]. The majority of reports regarding pancreas infection are based on immunohistochemical detection of enteroviral VP1 capsid protein [2,3,4], whereas only a few reports demonstrated viral RNA [2,3,5].

In fact, detection of EV RNA in the pancreas of T1D patients turned out to be challenging, and the amount of genome equivalents is extremely low and close to the detection limits of sensitive RT-PCR assays [2]. One reason for this can be the low number of infected cells, as only about 5% of the islet cells (that by themselves represent only 2% of pancreatic tissue) were found positive for the VP1 viral protein [6]. These findings suggest that the situation is not that of a typical active infection, but rather that of a persistent infection in which the virus replicates slowly in a limited number of cells producing low amounts of viral RNA. In some cases, this is due to terminal deletions in the 5′ UTR (untranslated region) of the enteroviral genome [7]. Current evidence indicates that, in mice, EV infection of heart, spleen, and pancreas can rapidly generate defective viruses that sustain loss of up to ~50 nucleotides at the 5′ end of the genome. Defective viruses characteristically result in persistent infections with a very low genome copy number in infected cells, whereas a normal virus with intact 5′ termini is eliminated [7,8,9]. The replication rate of this form of virus is greatly reduced due to a loss of 5′ hairpin structures that bind cellular host factors which stimulate the synthesis of both negative-strand and positive-strand RNA [10,11,12,13]. This type of infection is expected to poorly package the viral genome since virus assembly is directly linked to the replication rate [14]. Pancreas tissue contains compounds that may degrade free RNA such as unencapsidated virus forms [15], whereas encapsidated RNA within mature virus particles may resist pancreatic enzymes.

Thus, there is an intense interest in whether persistent infections as reported above can also be found in pancreata of T1D patients. In addition to the supposedly low virus amount, the pancreas may also contain compounds that inhibit PCR reactions. The above observations may explain why detection of EV RNA in the pancreas of T1D patients is so difficult. 

The nPOD (Network for Pancreatic Organ Donors with Diabetes) pancreas tissue biobank [16] greatly increases the possibility of studying human pancreata for a potential virus infection. The nPOD virus working group (nPOD-V) analyzed a large number of pancreas samples using both immunohistochemical and PCR approaches to substantiate virus detection. Results from nPOD-V indicate that RT-PCR is among the most sensitive methods to diagnose EV infection [17]; however, as described above, virus detection in the pancreas may be compromised by a number of factors.

We set out to evaluate the sensitivity of EV detection using different RT-PCR methods in pancreas homogenates. Particularly, we addressed the question whether pancreatic components may affect the ability of RT-PCR methods to detect encapsidated vs. unencapsidated virus RNA including the terminally deleted form of viral RNA. We also addressed if the organ procurement procedure (including transport to the laboratory on wet ice) could affect virus detection. This study was performed at three collaborating laboratories of the nPOD-V working group. Thus, we could compare the sensitivity of EV detection using five different RT-PCR approaches targeting the conserved 5′ UTR region of viral genome.

## 2. Materials and Methods 

### 2.1. Participating Virology Laboratories

Three virology laboratories of the nPOD-V consortium contributed to the study: (a) University of Tampere, Finland; (b) Baylor College of Medicine, Houston, Texas; (c) University of Insubria, Varese, Italy.

### 2.2. Preparation of Virus-Spiked Human Pancreas Homogenate (University of Tampere, Finland)

**Figure 1:**
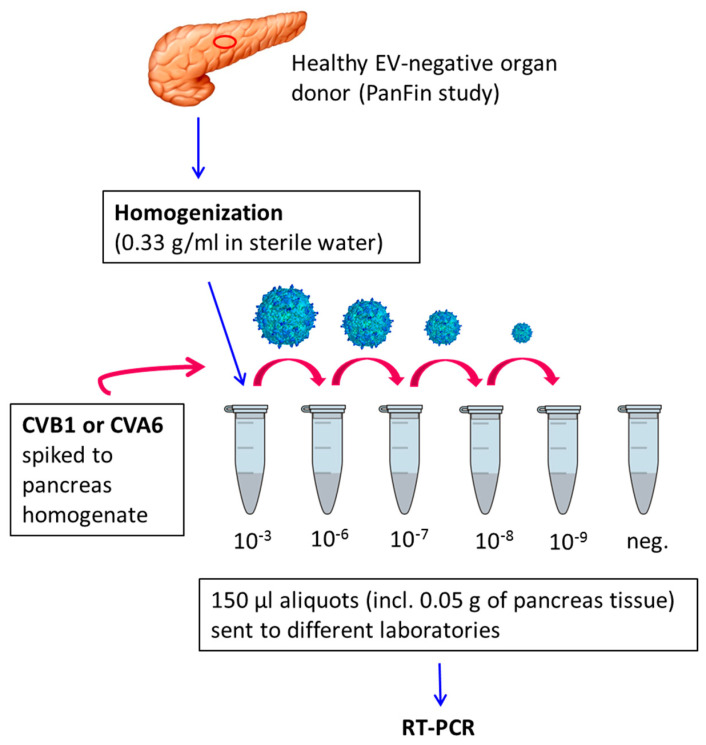
Overview of the preparation of pancreas homogenate dilutions spiked with coxsackievirus B1 (CVB1) and coxsackievirus A6 (CVA6).

An EV-negative pancreas sample of a non-diabetic organ donor from the PanFin study [18] was homogenized using a Silent Crusher S homogenizer (Heidolph, Schwabach, Germany). The pancreas extract was divided into aliquots and spiked with infectious virus preparations of either coxsackievirus B1 (CVB1) or coxsackievirus A6 (CVA6) (Figure 1). The virus strains were propagated in GMK (green monkey kidney) cells, and cell culture supernatant was used as virus source. Pancreas samples were spiked with virus dilutions (10^−3^, 10^−6^, 10^−7^, 10^−8^, 10^−9^) and immediately frozen at −80 °C. One set of CVB1 dilutions was kept on wet ice for 20 h after spiking and frozen as above. An identical CVB1 dilution series was made in sterile water. Pancreas extract and water samples with no addition of virus were used as negative controls.

Frozen samples were coded and distributed to the three laboratories. Blinded samples were tested at the three nPOD-V laboratories that used five different RT-PCR methods for EV detection. 

### 2.3. Virus Detection by RT-PCR

**Tampere laboratory**: RNA was extracted from pancreas homogenate samples using the Viral RNA Kit (Qiagen, Hilden, Germany). Samples were analyzed using two RT-PCR methods: a semi-quantitative RT-PCR coupled with hybridization with an EV-specific probe (PCR 1) [19], and a quantitative real-time RT-PCR (PCR 2) [20].

**Houston laboratory**: RNA was extracted with MagMax Viral RNA Isolation Kit (Invitrogen; ThermoFischer Sci., Waltham, MA, USA). RNA was converted to complementary DNA (cDNA) with Superscript III RT (Invitrogen) according to the manufacturer’s directions with random primers. PCR was carried out with SYBR-Green PCR master mix (Invitrogen) using the same primers as above [20]. PCR was carried out after a denaturation step of 95 °C for 10 min, followed by 50 cycles of 95 °C for 30 s and 60 °C for 60 s.

**Varese laboratory**: Pancreas homogenates were analyzed using two different procedures: (a) direct RNA extraction (PCR 1), and (b) RNA extraction following blinded passage in four different cell lines (AV3, RD, HEL-299, and LLC-MK2) in order to enrich the possibly present virus (PCR 2). The procedure was performed essentially as reported [21]. Total RNA was extracted from 0.6 mL of each sample (virus in water or in pancreas homogenates (PCR-1), mixed cell culture supernatants (PCR-2)) using an automated m2000sp instrument (Abbott Molecular, Rome, Italy). cDNA was produced with SuperScript III RT (Invitrogen) coupled to its master mix (random hexamer primers, RNase inhibitor, helper proteins). GoTaq DNA Polymerase master mix (Promega, Milano, Italy) was used for PCR reactions that were analyzed by electrophoresis in 1.5%–3% agarose gels containing GelRed (DBA, Segrate, Italy). Amplicons were sequenced using the Sanger method.

All used enterovirus primer pairs and probes are listed in Table 1.

### 2.4. Preparation and Detection of Full-Length and Terminally Deleted Coxsackievirus B3 (Baylor College of Medicine, Houston, Texas)

Infectious cDNA clones of full-length CVB3 strain 28 (CBV3), as well as of CVB3 containing a 5′ terminal deletion of 49 nt (TD-CVB3) [22], were used to transcribe RNA in vitro with T7 RNA polymerase in 60 min reactions. Transcribed RNA was purified with an RNA Clean/Concentrator kit (Zymo Research, Irvine, CA). The integrity of the transcribed RNA was confirmed by analysis on denaturing agarose gels, and RNA was quantified by ultraviolet (UV) spectroscopy. HeLa cells were grown on a 24-well plate overnight to a density of 1.2 × 10^5^ cells per well. Cells were transfected with a combination of the CVB3 RNA and plasmid DNA expressing GFP (green fluorescent protein) which served as carrier nucleic acid and as a marker of transfection efficiency. For each well, a combination of GFP plasmid DNA (250 ng) and 1.2 × 10^6^ genome copies of either CVB3 RNA or TD-CVB3 RNA was transfected using Lipofectamine 3000 (Invitrogen) and standard transfection protocols. This generated an estimated maximal transfection rate of 10 viral genomes per cell. GFP expression in control wells at 12 h post transfection was monitored by fluorescence microscopy, and the efficiency of plasmid/RNA uptake into cells was routinely judged to be approximately 80%.

Alternatively, cells were infected with CVB3 virus at MOI (multiplicity of infection) = 10. Transfected cells and infected cells were incubated for 6 h at 37 °C in the presence or absence of 2 mM guanidine HCl before cells were harvested. To remove unabsorbed transfection RNA or virus, cells were washed five times with growth medium, then treated with RNase A (1 mg/mL) in medium for 30 min at 37 °C. Cells were again washed three times with PBS (phosphate-buffered saline) and trypsinized. The transfected/infected cells were removed from the plate and mixed in serial one log dilution increments with untreated HeLa cells beginning at 10^5^ transfected cells to 10^5^ untreated cells, down to one transfected/infected cell in a background of 10^5^ untreated cells. Each cell dilution was centrifuged, the supernatant was removed, and pancreatic lysate was added to the cell pellets. Cells were incubated with pancreatic lysate on wet ice for 15 min, then flash frozen at −80 °C overnight before RNA extraction. RNA was isolated from cells in 50 µL of elution buffer using the MagMAX Viral RNA Isolation Kit (Invitrogen). cDNA was generated with the SuperScript III Reverse Transcriptase (Invitrogen) before analysis by qRT-PCR as described above.

## 3. Results

### 3.1. Sensitivity of Different RT-PCR Methods to Detect Enterovirus RNA

The ability of different RT-PCR methods to detect enterovirus RNA in pancreas was analyzed using pancreas homogenates spiked with different amounts of infectious CVB1 and CVA6 (Figure 1). The sensitivities of the RT-PCR methods showed variation, but all laboratories were able to detect even the smallest amount of virus in at least one sample type (Table 2). Although the methods were congruent and linear at high virus content, they became stochastic and less reproducible when copy numbers dropped to fewer than 1000 per sample. Two methods identified the virus even in the most diluted water sample, while three other methods were less sensitive. The sensitivity of different methods showed less variation in pancreas samples than in water samples. The incubation of pancreas samples on wet ice for 20 h had a minimal effect, where it reduced the sensitivity of two RT-PCR methods by one dilution step (10-fold difference), while the sensitivity of the three other methods was not affected by this 20-h pre-incubation phase.

### 3.2. Detection of Encapsidated and Unencapsidated Enterovirus RNA

**Figure 2:**
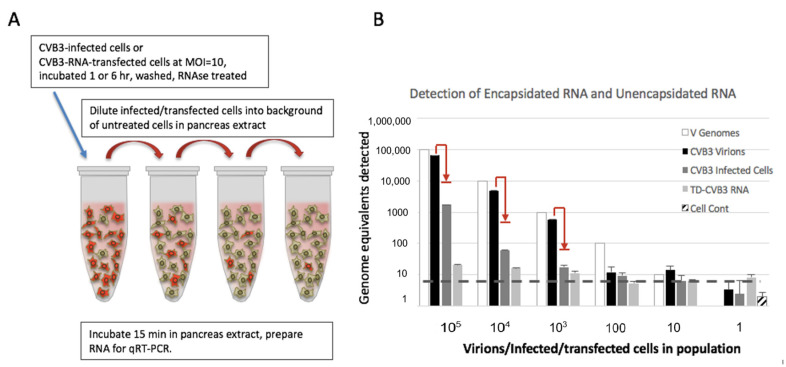
(**A**) The experimental procedure of diluting infected or cells transfected with viral RNA into a background of untreated cells and pancreas extract. (**B**) Comparison of recovery of three forms of viral RNA from in pancreas extract treatment. Intact CVB3 virions, CVB3-infected cells, or TD-CVB3 transfected cells across a range of dilutions of infected/transfected cells in a background of untreated cells were treated with pancreatic extract before RNA extraction and recovery. RT-PCR results are converted from Ct (threshold cycle) to genomes using a standard curve established with known quantities of viral RNA. RT-PCR assay with SYBR green has an effect on endpoint Ct, which, when converted on standard curves (dashed line), indicates an effective lowest level of detection in assay. Cell Cont represents assay results from untreated cells. The calculated input of virus genomes in each experiment is shown in white bars. The red arrows indicate expected loss of virus signal from virus not adsorbed by cells during the 1-h infection incubation and washing step.

To evaluate the ability of detecting unencapsidated viral RNA, an experiment was carried out where transfected cells containing viral RNA were diluted into a background of non-transfected cells before recovery from pancreatic extracts (Figure 2A). Using this approach, we firstly compared the sensitivity of the detection of encapsidated viral RNA (condition A) versus unencapsidated viral RNA inside the cells (condition B).

In condition B, cells were infected 60 min to allow uptake, penetration, and release into the cytoplasm. Unadsorbed virus was washed away, and infected cells were diluted into a background of uninfected cells (Figure 2A). Then, either virions (condition A) or infected cells (condition B) were incubated for 15 min on ice with pancreatic extract before extraction. Results (Figure 2B) indicate that nearly all input virion RNA can be recovered after incubation of CVB3 virions with pancreatic extract. This recapitulates data shown in Figure 1. However, much less unencapsidated virus RNA was recovered from infected cells when incubated with pancreas extract.

RNA from the wild-type CVB3, as well as free RNA purified from terminally deleted CVB3, was tested for stability in pancreatic extract after incubation on ice for 15 min. Results indicate that almost no such terminally deleted RNA survived the interaction with pancreatic extract for a short period of time (Figure 2B).

### 3.3. Detection of Replicating and Non-Replicating Enterovirus

**Figure 3:**
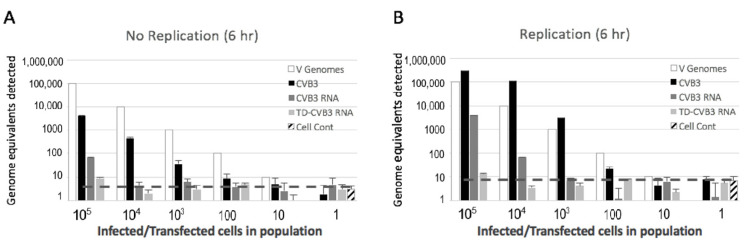
Detection of viral RNA from cells infected with CVB3 or transfected with equivalent genome copies of wild-type CVB3 RNA or truncated TD-CVB3 RNA. After infection or transfection for 6 h in the presence (**A**) or absence (**B**) of guanidine–HCl, the cells were diluted into non-transfected cells (as described in Figure 2A), treated with pancreas extract for 15 min on ice before extraction. RT-PCR analysis was the same as described for Figure 2B. Dashed line indicates effective limit of detection from standard curves and negative controls.

Since it is difficult to determine precisely how much transfected RNA is delivered into cells, we performed another experiment to compare viral recovery during replication versus non-replication conditions. Infections and transfections were carried out in HeLa cells as described above. Both wild-type CVB3 RNA and terminally deleted CVB3 RNA were transfected into the cells. To verify that viral RNA was introduced into the cells, we allowed six hours of incubation time for a measurable replication to occur (Figure 3B). Parallel sets of transfected cells were treated identically, except that virus replication was blocked by the addition of 2 mM guanidine–HCl (Figure 3A). As expected, results show a much higher recovery of virus RNA from CVB3-infected cells incubated without guanidine, indicating about a 17-fold increase in RNA from replication (Figure 3A,B). Similarly, recovery of RNA from the cells transfected with wild-type CVB3 RNA and incubated without guanidine was about 15-fold higher compared to cells incubated with guanidine (Figure 3A,B). This indicates that transfected viral RNA was delivered properly to cytoplasmic compartments to enable replication. However, the recovery and the detection of cytoplasmic viral RNA after transfection were much lower than those of RNA from natural virus infection, indicating that transfection delivery is less efficient.

Comparison of the detection of transfected wild-type CVB3 RNA versus terminally deleted CVB3 RNA from cultured cells indicates a further nine-fold loss of recovery under conditions where the replication was blocked (Figure 3A). There was little indication that terminally deleted CVB3 replicated in HeLa cells under these short time conditions (Figure 3A,B). The endpoint sensitivity of the detection of terminally deleted CVB3 was very poor under both conditions, with the detection above background occurring only when 10^5^ genome equivalents were introduced into the experiment. Notably, comparison of intact CVB3 RNA to terminally deleted CVB3 RNA indicated nearly a log lower detection level, likely due to the additional instability of terminally deleted CVB3 to cytoplasmic nucleases resulting from a loss of the stabilizing 5′ hairpin structure [23].

## 4. Discussion

The present study demonstrates that detection of encapsidated enterovirus RNA by RT-PCR was not much affected by a 20-h-long incubation on ice in pancreatic homogenates prior to RNA extraction, while even a short incubation on ice led to dramatic decrease in the detection of unencapsidated viral RNA. In addition, the results showed that the sensitivity of different EV RT-PCR assays differed slightly depending on the assay itself and the sample type analyzed.

All assays were capable of detecting very small levels of virus but did not always lead to amplicons when fewer than 1000 genomes per reaction were present, which is typical for this type of analysis. Importantly, the results indicate that pancreas tissue does not contain factors that significantly inhibit polymerase activity in molecular assays of this kind.

An additional test in the experimental design was to see if the transport of pancreas on wet ice, which is an unavoidable part of the nPOD organ procurement procedure, adversely impacts the recovery of RNA from encapsidated virions. The data indicate that it does not, as the 20-h incubation with pancreatic extract on wet ice did not cause a significant decrease in the number of viral genomes detected. Thus, the transport of pancreas tissues on wet ice seems to be adequate for EV detection by RT-PCR, preventing the loss of viral RNA which easily happens at high temperatures [24]. Together, these results indicate that there was a good concordance in the efficiency of virus detection among different laboratories and that pancreatic extract does not influence the stability of virions, nor does it inhibit the PCR methods used in the study.

Further experiments indicated that viral RNA with terminal deletions is rapidly destroyed by brief incubation on ice with pancreatic extract, and that detection of unencapsidated RNA was much lower than that of RNA encapsidated in complete virus particles. Under the conditions used, less viral RNA was recovered from transfected cells than from naturally infected cells. This could be partly due to the lack of nuclease-protective VPg (viral protein genome-linked) on transfected RNA and delivery via liposomes to cytoplasmic compartments that undergo lysosomal decay. These limitations make it difficult to quantitatively model the exact conditions of a low-level replicating terminally deleted CVB3 in cells. Nonetheless, the comparison of transfected RNA under non-replicating versus replicating conditions indicates that a proportion of transfected RNA is delivered to the correct cytoplasmic compartment to initiate genome replication. Under these conditions, we cannot precisely determine the limit of detection of unencapsidated CVB3 RNA in cells. However, taken together, the data indicate that detection would be approximately 100–1000-fold lower than that of encapsidated virion RNA. Detection of terminally deleted CVB3 RNA would add an additional eight-fold reduction of efficiency as compared to intact CVB3 RNA.

Taken together, the results indicate that virus RNA that is protected inside capsids is stable to the RNase activities in pancreas extract, such that no substantial effect on virus RNA detection was noted. However, an increasing amount of data suggest that virus in the pancreas of T1D patients may be terminally deleted and replication-deficient. Such virus may have a much lower rate of encapsidation since enterovirus encapsidation efficiency is coupled to replication rates [14]. Furthermore, the loss of stem loop A-B that binds cellular factor PCBP2 (poly(rC)-binding protein 2) decreases the stability of viral RNA to cellular ribonucleases [23]. Thus, the terminally deleted viral RNA that may be present in the pancreas of diabetic patients is not expected to be encapsidated, and it is likely less stable than virion RNA.

In conclusion, the study indicates that detection of unencapsidated viral RNA within rare cells in a population of uninfected cells, in an environment that contains pancreatic enzymes, is quite difficult. This does not derive from inhibitory effects of pancreatic factors on RT-PCR polymerases, but mostly from the exposure of viral RNA to RNase activities. The recovery of unencapsidated enterovirus RNA from such samples is not impossible but may require 100–1000 genome equivalents in each sample. The problem becomes more pronounced with terminally deleted EV that lacks a protective 5′ stem loop structure and undergoes encapsidation with greatly reduced efficiency [9]. The results should inform interpretation of future viral studies of human pancreas and other organs, such as the brain and the heart, where EV persistence is documented [25,26].

## Figures and Tables

**Table 1:** Enterovirus primer pairs used in different laboratories and in different methods.

Laboratory	Method	EV Group	Forward Primer	Reverse Primer	Probe	Location in the Genome *
Houston	PCR 1	5′UTR-A-D	CGGCCCCTGAATGCGGCTAA	GAAACACGGACACCCAAAGTA		449–563
Tampere	PCR 1	5′UTR-A-D	CGGCCCCTGAATGCGGCTAA	GAAACACGGACACCCAAAGTA	TAITCGGTTCCGCTGC	449–563
	PCR 2	5′UTR-A-D	CGGCCCCTGAATGCGGCTAA	GAAACACGGACACCCAAAGTA	FAM-TCTGTGGCG GAA CCGACTA-TAMRA FAM-TCTGCAGCGGAA CCGACTA-TAMRA	449–563
Varese	PCR 1 and 2	5′UTR-A	GTGTAGATCAGGTCGATGAGTCAC	ATTGTCACCATAAGCAGCCA		306–597
		5′UTR-B	GACCAAGCACTTCTGTTACCC	GTCACCATAAGCAGCCAATATA		161–594
		5′UTR-C	GGTGTGAAGAGCCTATTGAGC	GATTGTCACCATAAGCAGCCA		413–598
		5′UTR-D	TGGTCCAGGCTGCGTT	AACACGGACACCCAAAGTAGT		351–561

* Reference: EV68 GenBank accession no. AY426531; EV (enterovirus); UTR (untranslated region).

**Table 2:** Sensitivity of different RT-PCT methods to detect coxsackievirus B1 (CVB1) and coxsackievirus A6 (CVA6) in different sample types. The result indicates the most diluted sample where the RT-PCR gave a positive result.

	Houston	Tampere		Varese	
Sample	PCR 1	PCR 1	PCR 2	PCR 1	PCR 2
CVB1 in water	10^−7^	10^−9^	10^−9^	10^−6^	n.a.
CVB1 in pancreas extract	10^−8^	10^−9^	10^−9^	10^−9^	10^−9^
CVB1 in pancreas extract, 20 h on ice	10^−8^	10^−8^	10^−8^	10^−9^	10^−9^
CVA6 in pancreas extract	10^−7^	10^−7^	10^−7^	10^−8^	10^−9^
